# Fastq2vcf: a concise and transparent pipeline for whole-exome sequencing data analyses

**DOI:** 10.1186/s13104-015-1027-x

**Published:** 2015-03-08

**Authors:** Xiaoyi Gao, Jianpeng Xu, Joshua Starmer

**Affiliations:** Department of Ophthalmology and Visual Sciences, University of Illinois at Chicago, Chicago, IL 60612 USA; Department of Genetics, University of North Carolina-Chapel Hill, Chapel Hill, NC 27599 USA; Carolina Center for Genome Sciences, The University of North Carolina at Chapel Hill, Chapel Hill, NC 27599 USA; Lineberger Comprehensive Cancer Center, The University of North Carolina at Chapel Hill, Chapel Hill, NC 27599 USA

**Keywords:** Next generation sequencing, Whole exome sequencing, Variant calling, Pipeline, Automation

## Abstract

**Background:**

Whole-exome sequencing (WES) is a popular next-generation sequencing technology used by numerous laboratories with various levels of statistical and analytical expertise. Centralized databases, such as the Sequence Read Archive and the European Nucleotide Archive, allow data to be reanalyzed by independent labs to confirm results and derive additional insights. Access to new and shared data highlights the necessity for software that both lowers the statistical and analytical expertise required to generate results and promotes reproducible methodology among laboratories.

**Findings:**

We have developed fastq2vcf, a pipeline that automates the genomic variant calling process using multiple callers. Fastq2vcf offers improved flexibility, efficiency, and reproducibility by seamlessly integrating several leading sequencing analysis tools. It outputs not only the annotated variant call set for each caller, but also the consensus variant call set shared by different callers. Furthermore, it can be customized and extended easily.

**Conclusions:**

Our software tool automatically generates executable command lines for a variety of tools required for analyzing WES data. It is also highly configurable and provides users with complete control of the processing procedure, making it easy to submit and track jobs in both single workstation and parallelized computing environments. By using this pipeline, WES analysis can be easily reproduced.

## Findings

### Background

Whole-exome sequencing (WES) has been used by numerous biomedical researchers to identify disease markers and aid clinical decisions. The widespread deployment of different sequencing platforms; e.g., Illumina HiSeq/MiSeq/NextSeq, Ion Torrent PGM, Roche 454, Pacific Biosciences, and the SOLiD system, has made it feasible and affordable to carry out large-scale genomic studies using next-generation sequencing technology, such as WES. The most time-consuming part of WES data analyses is transforming the raw sequencing reads to called variants, which involves many specialized tools and repetitive steps. Many dedicated analysis tools require deep methodological knowledge [1], which presents a significant computational challenge, especially for small and mid-sized biology labs.

Although there are analysis pipelines publicly available that call similar popular next-generation sequencing (NGS) tools, such as FastQC, BWA, Picard, and GATK, they all have limitations that make them impractical in certain situations. HugeSeq [2] requires a highly specialized computing environment, i.e. Modules 3.2.8, Sun Grid Engine 6.2u2 and Simple Job Manager 1.0 (http://hugeseq.hugolam.com/documentation/requirements), which can be different from users’ computing facilities. Furthermore, setting up the computational environment can require the effort of a commercial team (https://www.sbgenomics.com/casestudies/stanford/). Web-based NGS tools, such as Galaxy [3], RUbioSeq [4], WEP [5] and STORMSeq [6], provide point-and-click tools to users, but are limited to the functions provided by the developers and using different or updated versions of tools, databases or reference genomes can be challenging. Moreover, sensitive data are not suitable to be transferred to third-party web servers. SIMPLEX [7] requires a special cloud computing setup for its server and client and also needs data to be transferred to web servers. Therefore, the development of a simple, transparent, and highly automated pipeline (hence easy to maintain, upgrade and customize) for variant calling analysis would be extremely useful for the bioinformatics community.

Here, we present fastq2vcf, a streamlined computational pipeline for fully automating the process of generating, annotating, and analyzing sequence variants. Moreover, the command lines generated by the pipeline are available to users and can be submitted directly in either a single workstation or a parallelized computing environment, which is different from all previous pipelines. Fastq2vcf is easy to use, since users only need to give the program a basic description of the sequence data. It is also flexible, as it allows all program parameters and command lines to be customized. Here, we use an example of WES to illustrate the implementation of the pipeline.

## Implementation

Fastq2vcf generates a comprehensive pipeline for fully automating the process of variant calling, from raw sequencing data in FASTQ format to called genomic variants in variant calling format (VCF), and their corresponding annotation. It is highly flexible and users can specify a variety of parameters for analyzing data according to the given biological problem.

**Figure 1:**
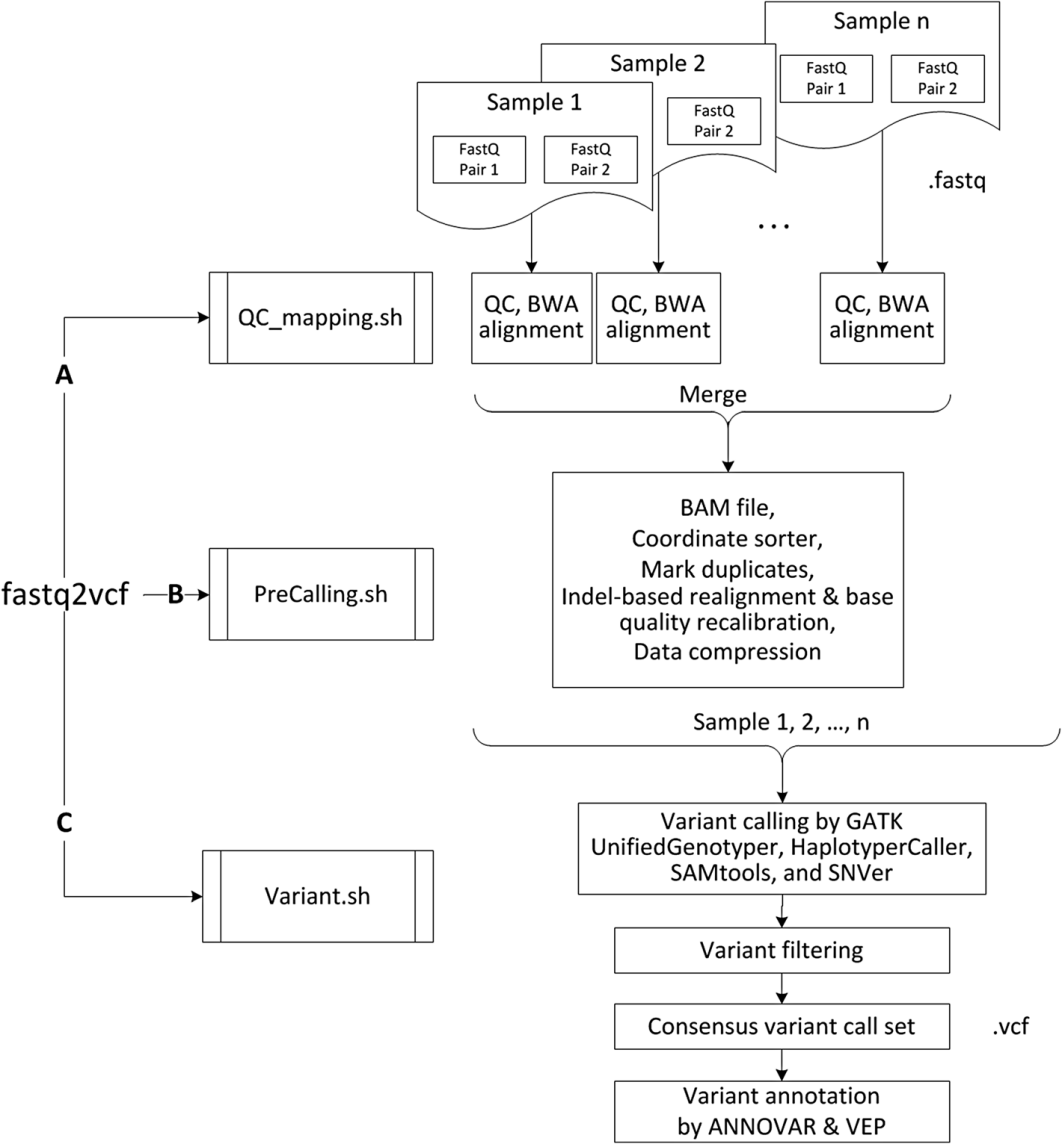
**Bioinformatics analysis workflow of fastq2vcf pipeline.**

Fastq2vcf requires two files: a data table describing the sequencing data and a configuration file, which are used to generate a series of shell scripts that can be run directly in a Linux/Unix environment. The sequencing data table contains information about sample identifiers, platforms, libraries, read groups, sequence types (Paired-End or Single-End), directories, and file names. Users can construct this table using spreadsheet programs or text editors and save it as a tab-delimited flat file. The configuration file stores the paths of the data analysis tools and program parameters. After configuring fastq2vcf, running it generates three categories of shell script files that automate all of the steps in the analysis pipeline. A typical pipeline is depicted in Figure 1, showing the output of fastq2vcf, three kinds of shell script files, and what these shell scripts do. The first, QC_mapping.sh, contains command lines for invoking quality control and alignment programs, and formats the data for further processing. The second, PreCalling.sh, contains command lines for removing duplicate data and for realignment to reduce false positives. The third script file, Variant.sh, contains command lines for variant calling, filtering and annotation.

## Results

### Quality control and sequence alignment

The first script in the pipeline (Figure 1A), QC_mapping.sh, contains command lines that invoke quality control (QC) and sequence alignment programs. The overall QC of the raw sequencing data is performed by FastQC (www.bioinformatics.babraham.ac.uk/projects/fastqc/). This program provides summaries of sequenced GC content, repetitive sequences, and many other potential anomalies, allowing users to evaluate whether the data have any quality issues. Alignment is then performed by BWA [8], the most common choice for variant calling analysis. The Sequence Alignment Map (SAM) file that BWA produces is then converted to the binary Alignment/Map (BAM) format using SAMtools [9].

### Marking duplicates, realignments, quality recalibration and data compression

Duplicate sequence reads are often generated from massively parallel sequencing instruments. These duplicates may produce a bias in estimating variant allele frequencies, and thus it is suggested to remove or mark them prior to variant calling. The second script that fastq2vcf generates (Figure 1B), PreCalling.sh, uses the Picard command-line tool, MarkDuplicate, to mark the duplicates with the FLAG field, which reduces the number of false calls and improves accuracy in the search for variants.

PreCalling.sh then employs the Genome Analysis ToolKit (GATK) realigner and the GATK recalibration tools to conduct local realignments and base quality recalibration [10,11], which helps to correct misalignments and systematic bias, and reduces false positives in variant calling.

### Variant calling, filtering and annotation

To carry out variant calling, the third script (Figure 1C), Variant.sh, invokes four variant calling programs: GATK UnifiedGenotyper, GATK HaplotypeCaller, SAMtools and SNVer [9,10,12]. These programs simultaneously detect both SNPs and INDELs and the results are reported in VCF files. Variant.sh then consolidates the outputs and reports the annotated variant call set for each caller and the consensus variant call set shared by all four callers.

The raw variant calls often include false positives that need to be filtered out. Thus, Variant.sh invokes the GATK tool VQSR (Variant Quality Score Recalibration) or Hard Filtering (for smaller datasets) [11] for performing variant refinement.

Lastly, Variant.sh calls both ANNOVAR [13] and VEP (ENSEMBL’s Variant Effect Predictor) [14] to annotate the called variants. ANNOVAR reports several annotations, including dbSNP IDs, gene annotations, variant function, allele frequencies from the 1000 Genomes Project, prediction scores of PolyPhen2 and SIFT, and many more. It generates a comprehensive variant annotation by conducting multiple levels of annotation analysis (gene-based, region-based, and filter-based). VEP provides similar function but sometimes can report different annotation from ANNOVAR [15]. A comparison between ANNOVAR and VEP has been reported by McCarthy *et al.* [15].

### Benchmarking

We tested fastq2vcf using a five-sample, 165 GB human WES dataset [16] downloaded from http://www.ebi.ac.uk/ena/data/view/SRP013517, on a Linux server with a dual Intel Xeon E5-2687 W CPU (3.10 GHz, 16 cores) and 256 GB of memory. The whole process took about 27 hours (QC_mapping 8 hours, PreCalling 8 hours, and variant calling by multiple callers and annotation 11 hours).

## Discussion

Recently, the NIH made plans to enhance reproducibility in the biomedical research community [17]. We believe that reproducibility in WES analysis comes from users’ transparent access to the actual command lines and the program parameters used. In response to the call for reproducibility in sequencing analysis, we designed a framework for WES that generates actual command lines (the same commands used to run WES manually), and stores them in files that retain a record of every step in the process. Thus, sharing the exact method used with another lab is as simple as attaching these files to an email. As far as we know, fastq2vcf is currently the only publicly available pipeline that generates command lines that can be shared so easily and submitted directly in either a single workstation or a parallelized computing environment. Furthermore, because the software does not run concurrently with the integrated WES analysis tools, it does not take any additional computing resources.

NGS is a complex and comprehensive research topic. It is unlikely for any pipeline to cover all options of the included tools and all kinds of situations in NGS. Hence, we made fastq2vcf easily customizable at several levels while keeping its design as simple as possible. If users need to use a different version of caller or reference genome, they can simply change the file path in the config file. If they need different parameters for the included NGS tools, this can be done three ways: changing the parameters in the config file, modifying the generated command lines (since these are the same command lines as users would type manually), or revising command lines in the fastq2vcf program. If users need to add a new tool, users can add several lines to fastq2vcf using our program as a template. For example, it took only three lines to add the VEP annotation function in fastq2vcf: 1) point to where VEP is stored at in config.ini; 2) retrieve the file path for VEP and 3) print out the VEP command line in fastq2vcf program. Lastly, we have hosted our pipeline in the Sourceforge Git repository and all interested users can participate in the software development. Since our pipeline generates actual command lines for NGS, it also serves as an educational tool to help novice users learn NGS analysis.

## Conclusions

We have developed fastq2vcf, an integrated analysis pipeline for WES data analysis, which offers improved flexibility, efficiency, and reproducibility. The fastq2vcf can generate shell scripts that automate the steps for processing WES data from raw sequence reads to annotated variants. This pipeline is also highly configurable and provides users with command lines stored in files that can be submitted directly in the Linux/Unix computing environment. This tool can be easily extended to include more analysis tools and customized for other types of NGS data analyses.

## Availability and requirements

**Project name:** fastq2vcf

**Project home page:**http://sourceforge.net/projects/fastq2vcf/

**Online users’ manual:**http://fastq2vcf.sourceforge.net

**Code home page:**https://sourceforge.net/p/fastq2vcf/code/ci/master/tree/

**Operating system(s):** Linux

**Programming language:** Perl, shell

**License:** GPL

**Any restriction to use by non-academics:** license needed
